# Variation in Elekta iView electronic portal imager pixel scale factor with gantry angle, and impact on multi‐leaf collimator quality assurance

**DOI:** 10.1002/acm2.13661

**Published:** 2022-06-06

**Authors:** Simon K. Goodall, Craig Norvill

**Affiliations:** ^1^ GenesisCare Wembley Western Australia Australia; ^2^ School of Physics Mathematics, and Computing Faculty of Engineering and Mathematical Sciences University of Western Australia Crawley Western Australia Australia

**Keywords:** DMLC QA, EPID, MLC QA

## Abstract

For Elekta Agility linear accelerators, the iViewGT electronic portal imaging device (EPID) is positioned at a nominal X‐Ray source‐to‐panel distance of 1600 mm. For display, image registration, and data processing purposes, the image pixels are scaled to spatial units at the treatment isocenter plane. This is achieved by applying a pixel scaling factor (PSF). During this investigation, the dependence of the PSF at cardinal gantry angles was determined along with the resulting effects on the multi‐leaf collimator (MLC) quality assurance (QA) results for three linear accelerators (linacs). The PSF was found to vary by 0.0018–0.0022 mm/pixel during gantry rotation, which resulted in variations in the mean MLC reported error of up to 0.8 mm at 100 mm off‐axis with the gantry rotated to 180°. Measurement and application of a gantry angle–specific PSF is a simple process that can be implemented to improve the accuracy of EPID‐based MLC QA at cardinal gantry angles.

## 1 INTRODUCTION

Volumetric modulated arc therapy and multiple gantry angle intensity‐modulated radiotherapy are commonly utilized in modern radiotherapy departments, and the requirement for an accurate multi‐leaf collimator (MLC) in these techniques is well documented.^[^ ^1^, ^2^, ^3^ ^]^ Multiple professional guidelines and publications recommend completing MLC quality assurance (QA) at multiple gantry angles to ensure the MLC performance is maintained over the full range of rotational motion.^[^ ^2^, ^3^, ^4^, ^5^, ^6^ ^]^

Most modern linacs are fitted with an electronic portal imaging device (EPID) that can produce images from the megavoltage (MV) beam and are an efficient tool for tasks such as MLC QA and patient‐specific quality assurance (PSQA).^[^ ^4^, ^5^, ^6^ ^]^ The Elekta Agility linear accelerator (Elekta, Stockholm, Sweden) is fitted with an EPID (PerkinElmer Inc., Santa Clara, CA) at a nominal source‐to‐panel distance (SPD) of 1600 mm. MLC position calibration, however, is typically defined at the isocenter plane. Therefore, the EPID image pixel dimensions at extended SPD must be converted to spatial units (typically millimeters) at the isocenter plane. This is achieved via a multiplicative application of a pixel scale factor (PSF).^[^ ^7^ ^]^

The PSF is derived by placing a calibration plate of known dimensions at isocenter, acquiring an image for gantry 0°, and relating the number of measured pixels to the known plate dimensions. For images exported to external software applications, the PSF may be entered manually as a software parameter or extracted from the image DICOM header information. The accuracy of this PSF, therefore, directly affects the accuracy with which MLC errors can be determined in physical space. It has been shown that the largest contribution to the variation in PSF between Elekta Agility linacs is derived from variations in the SPD.^[^ ^7^ ^]^ During rotation, the linac gantry head and EPID will flex due to rotational mechanics and the SPD may vary as a result, potentially introducing changes in the PSF.^[^ ^2^, ^6^ ^]^

This work aimed to investigate the variation in PSF with respect to gantry angle, and the effect on MLC QA results completed at different gantry angles. Finally, the study sought to investigate the level of consistency of this dependency across three linacs of the same model.

## 2 METHOD

Three Elekta Agility model linacs, each fitted with a 1024 × 1024 16‐bit pixel EPID (PerkinElmer Inc., Santa Clara, CA), were used during this investigation. Image acquisition was completed using the Elekta iViewGT (Elekta, Stockholm, Sweden) software v3.5 and a 6‐MV photon beam.

### 2.1 Pixel scale factor (PSF)

Before measuring the PSF, the linac isocenter lasers were confirmed to be coincident with the MV isocenter within a tolerance of 0.5 mm via a Winston–Lutz test.^[^ ^8^, ^9^ ^]^ The PSF was determined by capturing images of the Elekta beam limiting device (BLD) calibration plate, positioned at isocenter. The plate was positioned on the treatment couch and aligned using the linac crosshairs at gantry 0° and collimator 0°. The couch height was adjusted such that the coronal laser bisected the plate thickness. The field size was set to just cover the calibration plate, and a 20‐MU image was captured. The linac gantry was rotated to 180° and the image was repeated. To obtain images with the gantry at 90 and 270°, the calibration plate was attached to a 5‐cm thick plastic water slab so as to allow positioning perpendicular to the incident beam. The plate was again aligned to isocenter using the linac crosshairs and the sagittal laser.

The resultant images were processed using in‐house software (IHS) developed in MATLAB (MathWorks, Natick, MA). The functioning of this software and validation against the Elekta BLD calibration workflow have been described previously.^[^ ^7^ ^]^ The IHS returns the PSF, which allows the conversion of a measurement made in pixels to a physical distance at the linac isocenter. The PSF determined with the gantry at 0° is henceforth referred to as the nominal PSF.

### 2.2 Isocenter to panel distance

The vertical or horizontal distance between the relevant laser (isocenter) plane and the EPID front surface were measured using a steel ruler. Measurements were made at the center and each of the four corners of the panel surface, with covers removed. The average of these five measurements was reported as the isocenter‐to‐panel distance (IPD).

### 2.3 MLC QA

The Elekta AQUA MLC QA software (Elekta, Stockholm, Sweden) was used for subsequent testing of the MLC. A total of 23 EPID images were captured during an automated beam sequence delivery to assess MLC positions.^[^ ^10^ ^]^ The location of the MV isocenter is determined by finding the radiation center of nine 4 cm x 4 cm images at equidistant collimator rotations. Further images are acquired to account for collimator to EPID rotational offset. Images are then captured with the leaf banks positioned at five different off‐axis distances. MLC images are acquired at 0 and 180° collimator angles to capture all 80 leaves. For all measurements, the EPID panel is offset from isocenter by 13 cm. The position at the isocenter plane of each leaf in each bank is calculated relative to the MV isocenter location and the errors are reported.^[^ ^11^, ^12^ ^]^

For each of the three linear accelerators, the entire beam sequence was delivered to the EPID at all four cardinal gantry angles. This ensured a gantry angle–specific determination of the MV isocenter location. The resultant images were processed in the AQUA software using both the nominal PSF and gantry angle–specific PSF values. From these values, the changes in mean MLC error for each gantry angle and off‐axis leaf bank position were calculated for both nominal and gantry‐specific PSF.

## 3 RESULTS

For brevity, results are plotted only for the Y1 bank (X2 in IEC1217) of each linac. The symmetrical nature of the tests and geometric scaling of the PSF ensure the results from the Y2 bank display very similar results. Although the gravitational conditions vary for the Y1 and Y2 leaf banks when the gantry is at 90 or 270°, the results can be considered equivalent for opposed leaf banks under a 180° gantry rotation.

### 3.1 Pixel scale factor (PSF) and isocenter to panel distance

**FIGURE 1:**
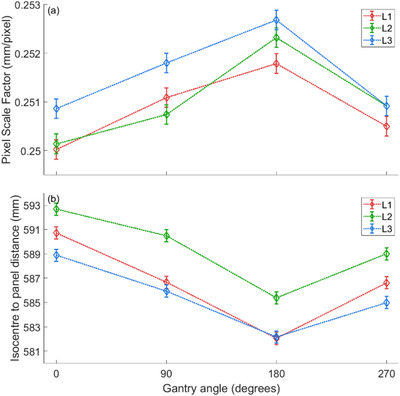
The variation in (a) pixel scale factor and (b) isocenter to panel distance with gantry rotation

The PSF was found to vary with gantry rotation, and each linac tested showed a similar pattern of change. As can be seen in Figure 1, the maximum PSF was determined with the gantry at 180° for each linac (L1, L2, L3). This corresponds to the maximum difference from the nominal value determined with the gantry at 0°.

As expected, the variation in IPD followed the same pattern as the variation in PSF. This variation in distance is the major contributor to the variation in PSF.^[^ ^7^ ^]^ The PSF was observed to vary by a maximum range of 0.0018 mm/pixel for L1 and L3, and 0.0022 mm/pixel for L2, respectively. This corresponded with maximum variations of 8.7‐, 7.3‐, and 6.7‐mm variations in the IPD for L1, L2, and L3, respectively.

### 3.2 MLC QA

**FIGURE 2:**
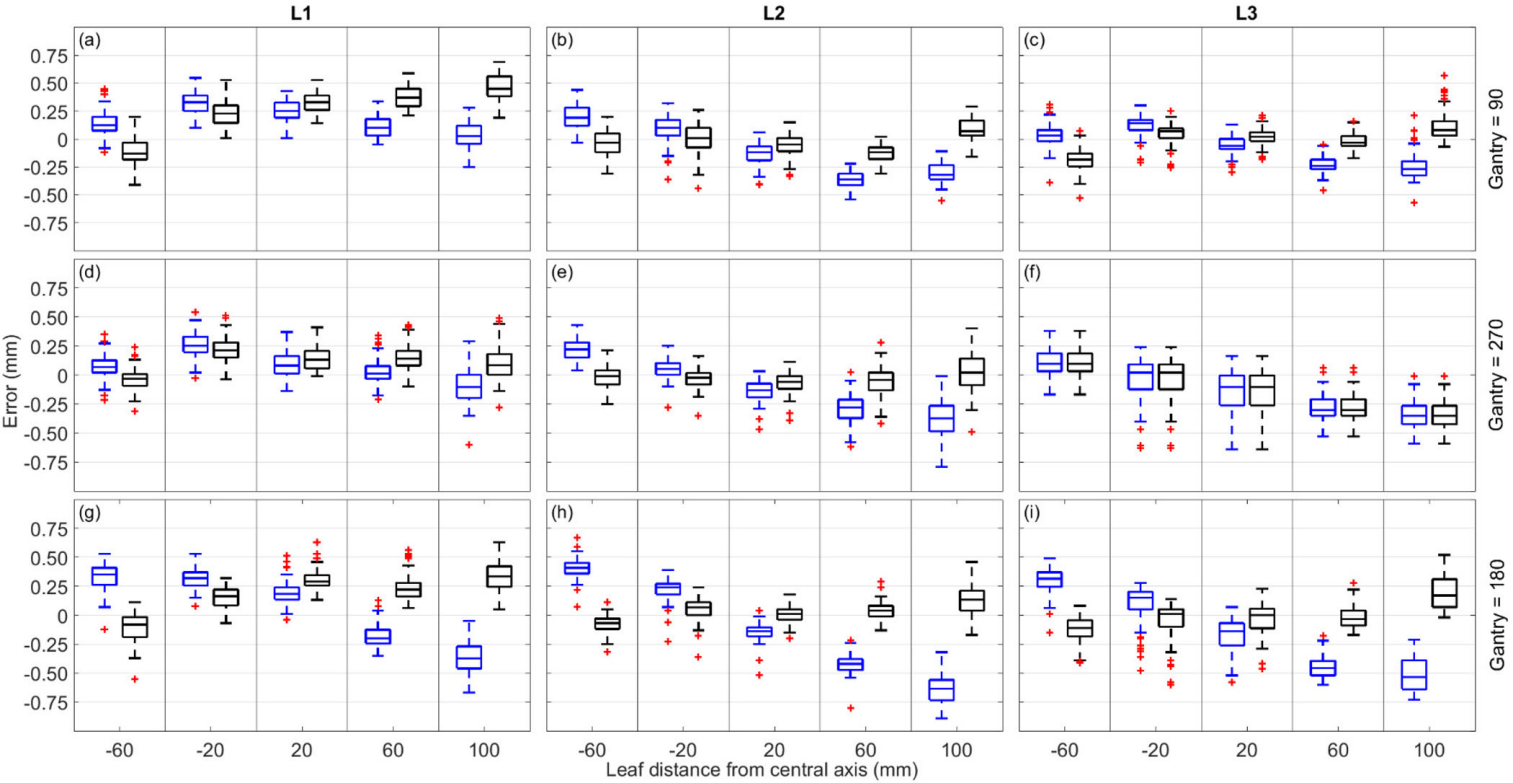
Boxplot of MLC positional errors at five distances from central axis. The top (a–c), middle (d–f), and bottom (g–i) rows show measurements with the gantry at 90, 270, and 180° respectively. Each column corresponds to a single linac. Each box represents the MLC errors when calculated using the nominal PSF (blue [first]) boxes, or the gantry angle–specific PSF (black [second]) boxes. MLC, multi‐leaf collimator; PSF, pixel scale factor

The change in MLC QA reported positional error, when measured at each gantry angle, and calculated using either the nominal or gantry angle–specific PSF can be seen in Figure 2. The change in determined MLC errors was observed to be largest when measured with the gantry at 180°, which is consistent with the PSF results in Figure 1. The impact of gantry‐specific PSF on MLC position error additionally increases with distance from the central axis, a direct result of the PSF being a scaling factor.

For a given linac, the PSF values observed in Figure 1 for measurements at gantry 90 and 270° are reasonably similar. As a result, the variations in measured MLC errors at these gantry angles are similar when alternating between the nominal and gantry angle–specific PSF. This further suggests the dominant cause of the PSF change is the variation in SPD due to gravitational effects of EPID sag parallel to the beam axis.

**FIGURE 3:**
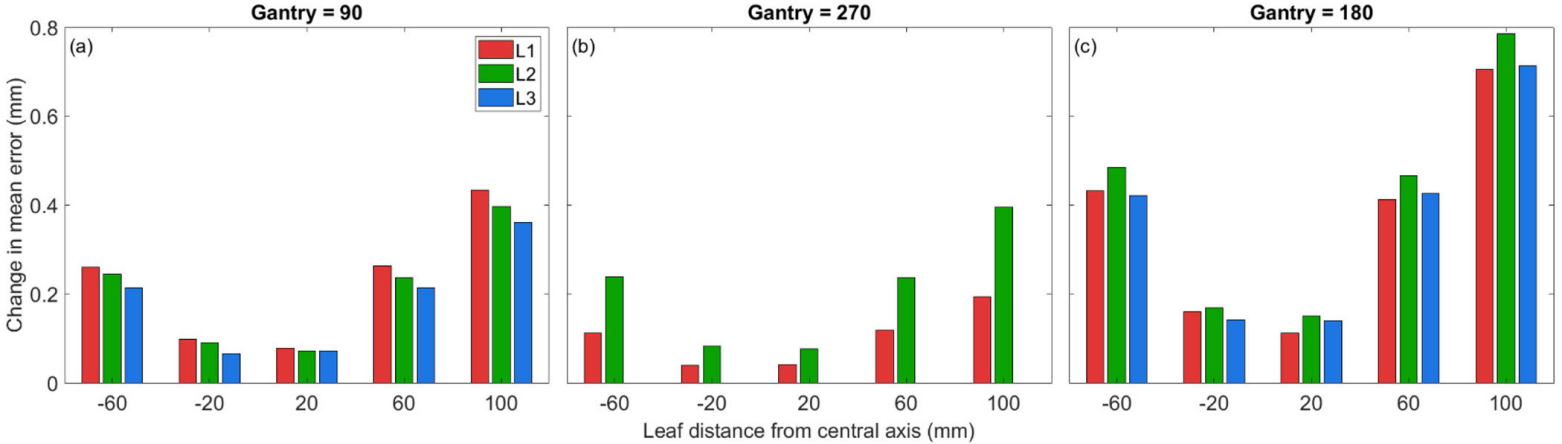
Change in mean absolute error of MLC bank position when converting from a nominal PSF to a gantry‐specific PSF at (a) Gantry = 90° (b) Gantry = 270° and (c) Gantry = 180°. MLC, multi‐leaf collimator; PSF, pixel scale factor

The change in the mean absolute error of the MLC bank at each position, for each linac, and each gantry angle can be seen in Figure 3. Each linac showed a similar pattern of dependence for MLC error after application of a gantry‐specific PSF, with small changes in the absolute magnitude observed.

Maximum changes in the mean error of 0.4 mm were observed for gantry angles of 90, 270°, and 0.8 mm for a gantry angle of 180°. Each of these maximum observed values was for leaves positioned 100 mm from the central axis. When considering inter‐linac variation, the maximum changes in mean absolute error were observed to be 0.7, 0.8, and 0.7 mm for L1, L2, and L3, respectively. Each of the maximum values occurred with the gantry at 180°, and the MLC at the furthest off‐axis position tested. For L3, the nominal PSF was equal to the PSF obtained with the gantry at 270°.

## 4 DISCUSSION

During this investigation, the SPD was observed to change during gantry rotation, resulting in a variation in calculated PSF values at different gantry angles. The pattern and magnitude were similar across three linacs of the same model. The variation in PSF relative to gantry 0° was most significant with the gantry rotated to 180°, resulting in the greatest change in measured MLC position. The results observed with the gantry at 90 and 270° were comparable and only showed a small to negligible change from the nominal PSF. This indicates the PSF follows a near symmetric pattern during rotation and suggests an equally balanced gantry/EPID at opposing angles. Due to the PSF function as a scaling value, the effect of applying a gantry‐specific PSF to measured leaf position increases with distance from the collimator central axis.

The magnitudes of change in mean MLC error for measurement points within 20 mm of isocenter (field sizes ≤40 × 40 mm^[^ ^2^ ^]^) were always less than 0.2 mm. This result shows the variation in small field sizes is minimal, which is important given small fields have been shown to be those most affected by changes in MLC aperture size and are relevant to techniques such as stereotactic radiosurgery (SRS) or stereotactic body radiotherapy (SBRT) where high‐quality MLC calibration is of extreme importance.^[^ ^2^, ^13^, ^14^ ^]^ For MLC positions offset from the beam axis by ≥100 mm, changes in the mean MLC position of 0.4–0.8 mm were observed. The changes in the MLC position observed at greater distances from central axis could be considered clinically important given MLC QA tolerances are typically ≤1.0 mm,^[^ ^2^ ^]^ and modern treatments can utilize isocenters offset from the beam central axis. This is especially the case in high‐end modern techniques such as single‐isocenter multiple‐target SRS.^[^ ^15^ ^]^

For measurements made with the gantry at 90° or 270°, maximum changes in the mean MLC position were observed to be less than 0.4 mm, reducing to 0.3 mm when considering positions within 60 mm of the central axis only. The magnitude of these changes is small and may not result in clinically significant changes to treatment plans during conventional radiotherapy, but may be of importance for SBRT or SRS. With the gantry at 180°, the mean change in MLC error ranged up to 0.8 mm. This is likely to be considered of importance against recommended published tolerances.

It is shown in Figure 2 that the implementation of a gantry angle–specific PSF does not always result in a reduction of measured error for the MLC positions. As a result, ensuring that the MLC is within a specified tolerance when using a nominal gantry PSF does not ensure that the MLCs are within the same tolerance when using a gantry angle–specific PSF.

MLC calibration is typically performed at a gantry angle of 0°. The purpose of MLC QA at other gantry angles is to ensure performance remains within a given specification over the full rotational range. When using an EPID for MLC QA, an application of the nominal PSF at other angles could potentially lead to inaccurate test results. The use of methods that do not rely on the EPID such as film may be an alternative MLC QA solution for these gantry angles; however, they lack the ease and efficiency of EPID‐based methods.^[^ ^10^ ^]^ Further work is required to investigate the changes in EPID‐based PSQA results that could be expected from implementing a gantry angle specific PSF; however, from these results it could be suggested that plans that deliver a high proportion of the dose from posterior gantry angles could show changes in PSQA results, depending upon the complexity of the treatment and the tolerances applied.

## 5 CONCLUSIONS

The PSF was found to be a gantry angle–specific value for Elekta Agility linacs, and when implemented resulted in changes to the mean recorded MLC errors by up to 0.8 mm. Measurement and application of a gantry angle–specific PSF is a simple process that can be implemented during EPID‐based MLC QA of cardinal gantry angles.

## CONFLICT OF INTEREST

The authors declare that there is no conflict of interestthat could be perceived as prejudicing the impartiality ofthe research reported.

## AUTHOR CONTRIBUTIONS

Simon K. Goodall conception and design of the project, acquisition of data (∼50% of final data), analysis and interpretation of the data. Drafting the manuscript and production of the figures. Final approval of manuscript.

Craig Norvill conception and design of the project, acquisition of data (∼50% of final data), review of analysis and interpretation of data. Critical review and editing of manuscript figures. Approval of final manuscript.
